# On the interdependence of insertion forces, insertion speed, and lubrication: Aspects to consider when testing cochlear implant electrodes

**DOI:** 10.1371/journal.pone.0295121

**Published:** 2024-01-24

**Authors:** Max Fröhlich, Daniel Schurzig, Thomas S. Rau, Thomas Lenarz

**Affiliations:** 1 MED-EL Medical Electronics GmbH, MED-EL Research Center, Hannover, Germany; 2 Department of Otolaryngology, Hannover Medical School, Hannover, Germany; 3 Cluster of Excellence EXC 2177/1 “Hearing4all”, Hannover, Germany; KIST: Korea Institute of Science and Technology, GERMANY

## Abstract

**Objectives:**

During the insertion of cochlear implant (CI) electrode arrays, forces occur which may cause trauma and poorer hearing outcomes. Unfortunately, research groups investigating factors influencing insertion forces come to contradicting results, especially regarding insertion speed. This study was conducted to investigate the origin of these contradicting results and to determine how different testing conditions influence experimental findings.

**Methods:**

Repeated, automated insertions with three different FLEX28 CI electrode arrays (MED-EL, Innsbruck, Austria) were performed into a newly developed, anatomically correct and 3D-printed mean scala tympani phantom. The testing protocol for each electrode included variations in insertion speed (v = 0.1–2.0 mm/s) and lubrication (90%, 50%, and 10% liquid soap), resulting in 51 insertions per electrode array and a total of 153 insertions.

**Results:**

The test setup and protocol allowed for repeatable insertions with only minimal change in the morphology of the insertion force profiles per testing condition. Strong but varying dependencies of the maximal insertion forces and work were found regarding both lubrication and speed: work-speed dependency is constant for the 10% lubricant, negative for the 50% lubricant and positive for the 90% lubricant.

**Conclusion:**

Our results can explain part of the contradicting results found within previous studies by translating interrelations known from lubricated rubber friction to the field of CI electrode array insertion. We show that the main driver behind measured bulk forces are most likely the generated friction forces, which are strongly dependent on insertion speed and lubrication. The employed test setup allows for conducting repeatable and comparable insertion studies, which can be recapitulated by other centers due to the detailed explanation of the test setup as well as the developed and freely available insertion phantom. This study hence represents another important step toward standardizing CI array insertion testing.

## Introduction

Cochlear implant (CI) surgery is a procedure to restore hearing in patients with severe to profound sensorineural hearing loss [1]. In this intervention the electrode array of the CI is inserted through the round window (RW) or cochleostomy [2] into the scala tympani (ST) of the cochlea [3]. Atraumatic surgical techniques [4] are applied to preserve the refined intracochlear structures and thus residual hearing, which can provide significant benefits in speech perception, e.g. by allowing for electric-acoustic stimulation [5].

In order to further improve CI hearing preservation outcomes either by advancing manual surgical techniques, optimizing electrode array design or developing surgical assisting systems [6–8], the insertion behavior of CI electrode arrays has been studied ever since the early days of CI development. Human cadaver temporal bone studies were a first attempt to investigate the interaction of electrode array and cochlear structures during insertion [9, 10]. The outcomes of these investigations were used to derive a scale for to quantifying electrode insertion trauma [11]. Later, insertion forces were measured in cadaver specimen to quantify the interaction of electrode array and intracochlear structures [7, 12, 13], and several artificial insertion phantoms were developed to better understand insertion dynamics [14–19].

While artificial insertion phantoms do not accurately represent the complex viscoelastic mechanical properties of living human tissue [20], these phantoms can be employed for large numbers of insertions in a controlled environment and are excellent models for studying basic influences and conducting parameter variations. However, results between groups are difficult to compare as there is no standardized approach for testing with physical phantoms, e.g., regarding geometry, manufacturing process, material and lubrication. Hence, findings vary substantially even if exclusively focusing on long, flexible lateral wall arrays such as the FLEX28 or STANDARD electrode (MED-EL, Innsbruck, Austria) or corresponding dummies: some groups reported an increase in maximum average insertion forces with increasing insertion speed [21–23] while others found the opposite interrelation [24]. Yet again, some groups report no speed dependency of insertion forces [25]. For automated insertions, some groups had difficulties with repeatability of their experiments, resulting in a maximum force variance of >60% [24] for consistent testing conditions or issues with consistent, complete electrode array insertions especially at lower insertion speeds [21]. On the other hand, some groups report successful insertions in all cases and a very good repeatability [22, 26]. Furthermore, when measuring insertion forces with the force sensor located above the electrode [25, 27–29] or below the insertion phantom (e.g.[7, 12, 13, 17, 21, 22, 24, 26, 30, 31]), typically only the bulk force behavior of this complete mechanical system is measured.

The main components in the mechanical system of the reported force measurement setups include the following: (I) the *electrode* (array and lead), (II) the *scala tympani* or *insertion phantom* and the fluid filling the ST, acting as a (III) *lubricant*. Several factors have a direct impact on the behavior of each one of the system components as well as on their interaction during insertion, most likely contributing to the contradicting results between groups. Those factors can be categorized into rigid boundary conditions and resulting dynamic behavior. The rigid boundary conditions are the *test setup* (i), and in phantoms the *interacting surface area* (ii) of the ST defined by its geometry and material. Both of these factors directly influence the *dynamic viscoelastic insertion behavior* (iii) of the electrode as well as the *dynamic mechanical interaction* (iv) of electrode, lubricant, and ST phantom.

The influence of the general *test setup* (i) and protocol should not be underestimated either: it was shown that by controlling and varying the insertion trajectory into the ST, mean maximum force values can vary by as much as 60% [22]. When aiming at studying the interaction of electrode array and the cochlear anatomy, the insertion phantom *surface area* (ii) should be anatomically correct representations of the ST geometry. It is, for instance, known that the complex geometry of the spiral ligament at the lateral wall changes along the cochlear spiral [32–34], which has an impact on the contact area between straight electrode arrays and the lateral wall [35]. However, several ST phantoms proposed within previous studies do not accurately represent these anatomical properties, either due to limitations of the manufacturing process [14, 21] or to the fundamental design of the model [15, 16]. Furthermore, there are characteristic ST properties (i.e. shape, length, cross sectional area) that can vary substantially between individuals [34, 36–38]. This is highly relevant, as was shown by Dhanasingh et al. [39], who showed that smaller sized ST phantoms yield higher insertion forces across different electrode array lengths. Those results are consistent with findings of Aebischer et al. who developed 6 geometrically highly accurate insertion phantoms from segmented ST data of individual cochleae [17]. Based on these models they were able to demonstrate the interdependence between cochlear size and insertion force magnitudes [22]. This is further supported by previously reported findings regarding force dependence on anatomical parameters [14] and the study of Hrncirik et al. who recently showed that insertion forces depend on ST size and curvature with respect to insertion depth of the electrode [18]. It could hence be argued that if investigating fundamental CI insertion phenomena, a physical phantom based on an average representation of the human ST might be better suited than individual models.

The *viscoelastic behavior of the electrode* (iii) is driven by its wire composition, as they cause differences in stiffness and elasticity, and hence bending behavior [22, 40]. Zuniga et al. suggested that extracochlear electrode deformation may impact insertion forces, as electrode buckling caused > 50% of incomplete insertions for faster insertion speeds in their setup [21]. Furthermore, it is known that test sample conditioning in repeated dynamic testing causes large deviations in results [41], especially in case of rubber composite materials like CI electrode arrays.

The *dynamic interaction of electrode*, *lubricant*, *and ST phantom* (iv) can be characterized by both forces from fluid and solid mechanics. Fluid mechanical forces occur as the electrode array must displace the fluid in the filled lumen during its insertion. Those findings were verified for CI electrode insertions as Todt et al. reported a relationship between static intracochlear fluid pressure and insertion speed [42], and more recent data shows that this relationship is statistically significant [43]. Tangential friction forces from solid contact mechanics are a result of the electrode array sliding along the lateral wall during insertion. Hence, friction forces increase as the array moves deeper into the ST [22, 26, 44]. It has also been shown in previous studies on the sliding behavior of rubber tires that the friction coefficient of rubber is not constant [45, 46]. Furthermore, it has been shown that there is a strong interdependence of the contact area between two frictional bodies, the lubricating fluid and friction forces [45, 47]. Those findings are complemented for silicone rubber electrode arrays as varying friction coefficients for different lubrications and different sides (hence, contact areas) of the same array [44] or other geometrical shapes [48] are reported. A dependence of friction coefficient and insertion speed has also been shown to have an impact on insertion force [25]. Furthermore, Kontorinis et al. reported an effect of lubrication on insertion forces [27].

Motivated by the described results within previous literature on CI insertion testing, the present study was conducted to achieve two objectives: firstly, a test setup and protocol had to be created which allow for highly repeatable insertion testing. This included the derivation of an anatomically correct physical insertion phantom based on an average representation of the human ST to preserve all common anatomical ST features which may influence CI insertion forces. The presented descriptions of the testing conditions and protocol as well as the free availability of the derived ST phantom are supposed to enable other centers to conduct comparable, standardized experiments in the future. The second goal of the present study was to conduct a series of CI insertions and to employ mechanical interaction phenomena known from the field of lubricated rubber friction to explain the effect of different CI insertion speeds and perilymph substitutes inside the ST insertion phantom onto resulting insertion forces.

## Materials and methods

### Scala tympani mean insertion phantom

**Fig 1:**
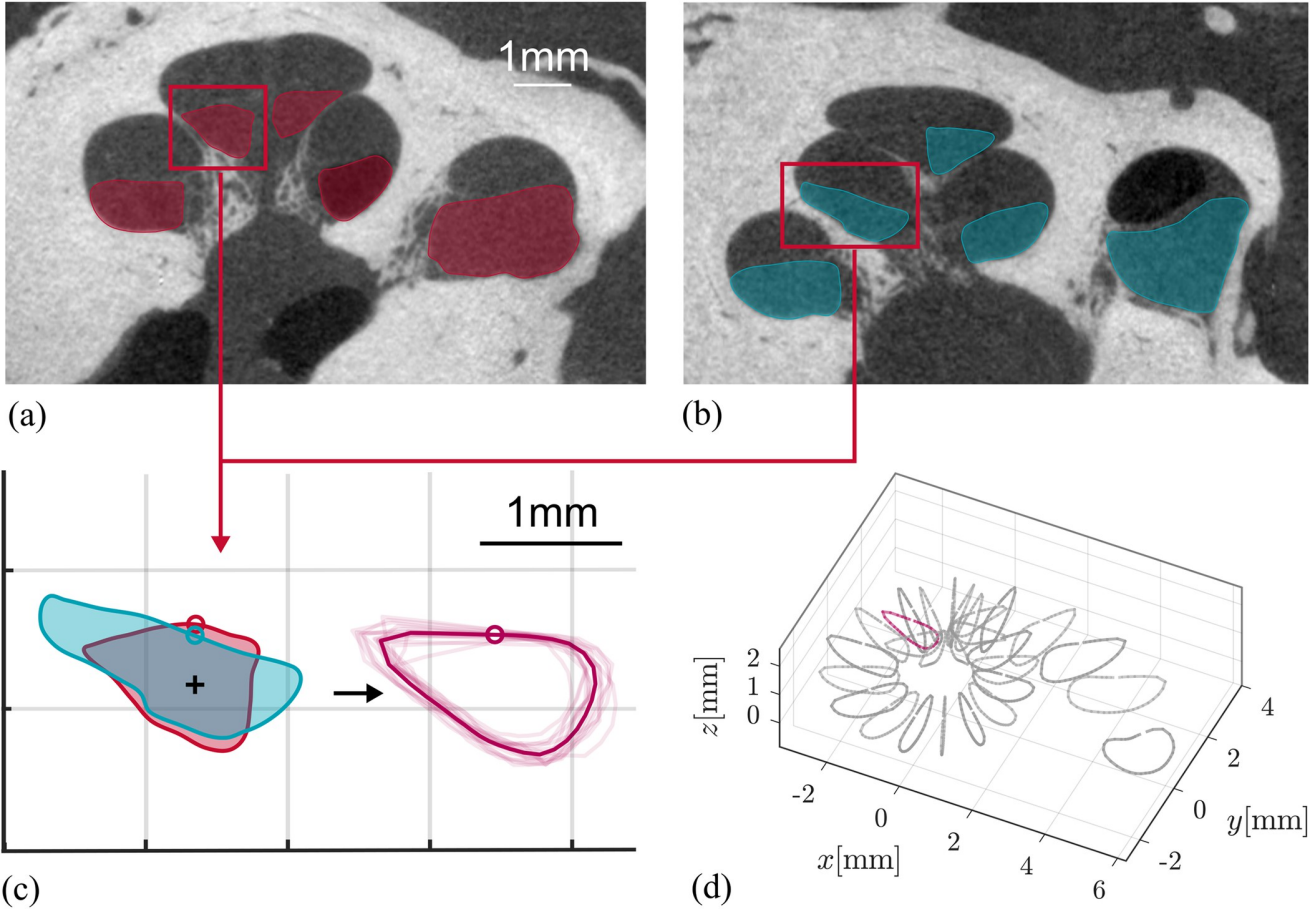
Scala Tympani (ST) model generation. Generation of the ST model follows several steps to preserve the common anatomical features of the cross-sectional geometry *[51]*, as this defines the contact area between electrode and phantom. Cross sections can vary in size and orientation between individuals (a,b) *[3, 34]* and were rotated to the cochlear angle dependent average orientation of the basilar membrane (c). Then manual segmentations points were redistributed evenly and consistent for all cross sections of the n = 15 datasets and datapoints were averaged at the respective angular location (d).

The data used for ST phantom generation was previously presented in earlier studies from our group [34, 49]. From 15 human micro-CT datasets, cross sections of the ST and other structures were manually segmented every 22.5° using a custom software tool designed for this task [50]. The angular step size was chosen since previous research showed that interpolation of cochlear cross-sections which are more than 45° apart may yield falsifications of the interpolated anatomy [49]. The method to create an anatomically correct average spiral ST representation of those 15 datasets was presented in [51], as several steps are necessary to preserve common anatomical features of the cross-sectional geometry: The manual segmentation points for each cross section were redistributed evenly and in relation to the centroid of each area. This consistent redistribution throughout all datasets is essential for accurate averaging. As cross-sectional geometries can vary largely in shape and rotation in between individuals (Fig 1A and 1B), this process allows for maintaining the qualitative shape of the ST without falsification. Furthermore, cross sections were rotated to the cochlear angle dependent average orientation of the basilar membrane before point redistribution and averaging (Fig 1C). The described procedure was performed for all cross sections of the 15 ST segmentations at the respective angular location (Fig 1D). Cross sections were interpolated for every 10° along the spiral to create the virtual ST model. All processing of these segmentations was done in Matlab (version R2018a, MathWorks, Natick, MA, USA).

**Fig 2:**
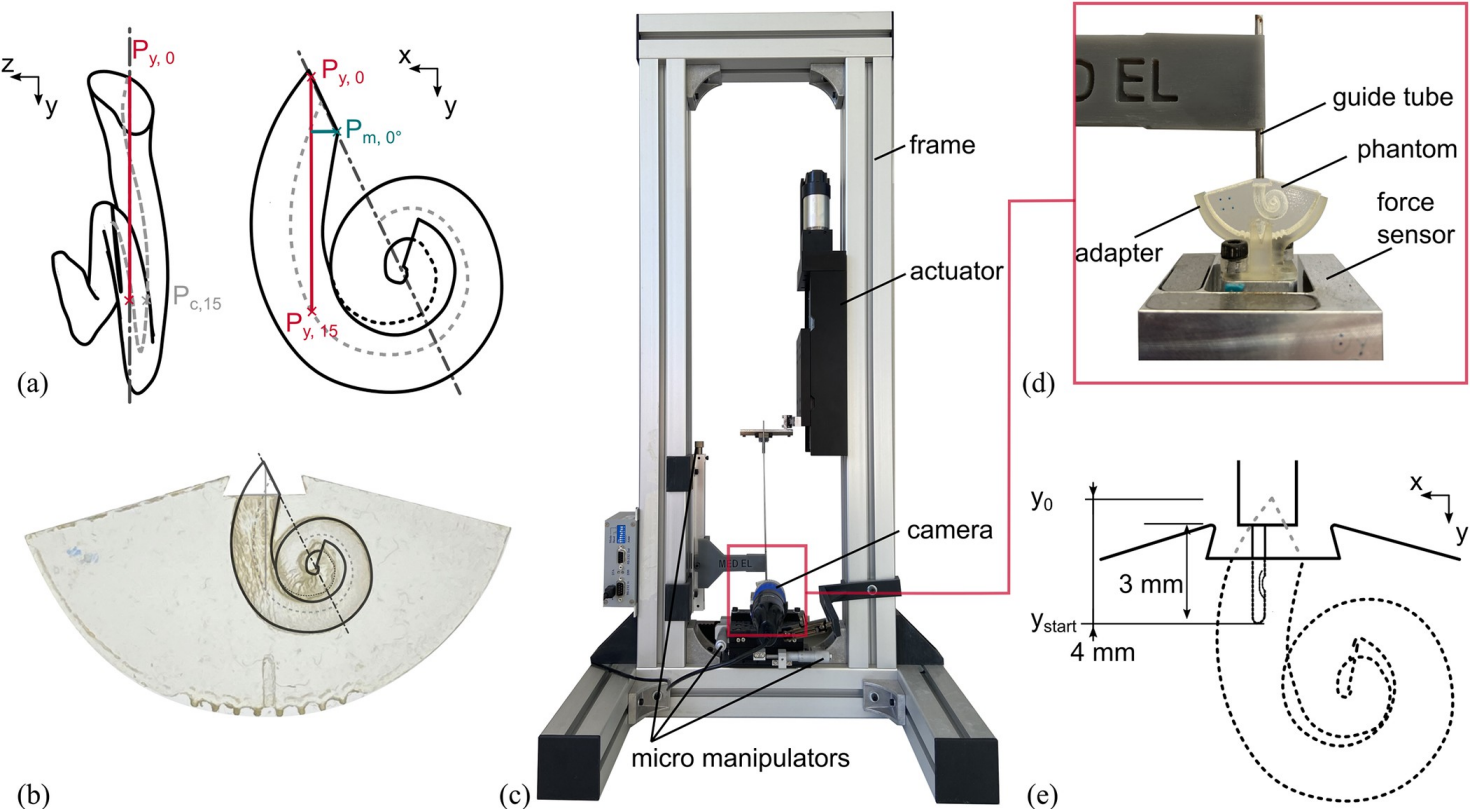
Phantom orientation and test setup. For the orientation of the ST model (a) the CCS [52] is adopted with the y-axis being the insertion axis. The model is transferred to the physical insertion phantom (b) and an idealized cochlea opening is created. The model is placed in the test setup (c) for CI electrode array insertion. To control the boundary conditions and create a steady state during insertion, the electrode array is inserted through a guide tube (d) into the phantom. Electrode insertion starts at EID_4_ = 4mm = y_start_ (e).

For a standardized placement of the average ST model inside in the phantom frame, an insertion coordinate system (ICS) was defined following the consensus cochlear coordinate system (CCS) [52], i.e. with the z axis of the model coinciding with the mid-modiolar axis of the ST (Fig 2A). For defining the y axis, the auxiliary point P_c,15_ was computed according to [53], i.e. as the location at which the gravitational centerline of the ST shifts by more than 15°/mm. The line from the center of the RW to this point was then projected onto the xy plane, yielding the y axis. Finally, the x axis was defined as orthogonal to y and z (see S1 Appendix for further information on translation vector and rotation angle).

In order to create the cochlear phantom, the average ST model was placed in the body of the phantom frame such that the round window region was cut at the most modiolar point P_m, 0°_ of the RW cross-section to create an ideal cochleostomy opening (Fig 2B). As the aim of the study was to measure intracochlear forces, this idealized opening was performed to facilitate electrode insertion along the trajectory of the longitudinal axis of the basal turn [54] and to avoid measuring contact and friction forces at the round window [22]. Hence, the insertion trajectory into the phantom can be described as a 0° angle in both the mediolateral (in-plane) and basoapical (out-of-plane) direction. The rounded contour at the bottom of the phantom allows for the phantom to be rotated inside the adapter for variation of insertion trajectories in future studies. Undercut features are located on both sides of the cochleostomy opening and can be used to mount different round window geometries onto the phantom. A 1 mm diameter pressure release hole was created in the apex of the ST geometry to allow for lubricants to flow out of the model hence suppress fluid pressure build up. A parametric description of the mean ST can be found in Table 1. A_ST_ and B_ST_ represent the basal diameter and width respectively [49]. H_ST_ represents the total height and H_S, ST_ the height of the lateral wall spiral of the mean ST model [55]. The cochlear duct length CDL_LW, ST_ is measured at the lateral wall of the ST.

**Table 1:** Parametrization of mean insertion phantom. The parameters A_ST_ (basal ST diameter, measured from the center of the round window through the modiolus to the opposite wall of the ST) and B_ST_ (basal turn width of the ST orthogonal to A_ST_) describe the dimensions of the basal turn of the mean ST. H_ST_ (distance from the lowest to the highest point f the ST lumen along z) describes its overall height and H_S, ST_ the height of its lateral wall (LW) spiral [49]. The Cochlear duct lengths CDL_LW,ST_ and CDL_ST_ are measured along the LW of the phantom from the center of the round window to the most apical point and express the corresponding length as metric and angular length respectively [55]. The parameter “Area cochleostomy” describes the surface area of the basal ST phantom opening for CI array insertion.

A_ST_	B_ST_	H_ST_	H_S,ST_	CDL_LW, ST_	CDL_ST_	Area cochleostomy
[mm]	[mm]	[mm]	[mm]	[mm]	[deg]	[mm^2^]
9.53	6.85	3.24	1.72	32.9	675°	2.19

The phantom was produced by an Aiglista 3D printer (Keyence, Osaka, Japan) with a resolution of 15 μm step size. Materials used were a transparent acrylic UV curing PUR solution and a polypropylenglycol based, water solvable support material (AR-M2 and AR-S1 respectively, Keyence, Osaka, Japan).

Both, the average model of the human ST and the insertion phantom can be downloaded at https://vianna.de/acms.html.

### Electrode insertion test setup

The main components of the test rig used for the present study are a linear actuator (type M-413, Physik Instrumente (PI) GmbH & Co. KG, Karlsruhe, Germany) to move the electrode array (Fig 2C) as well as a 3D force sensor (type K3D40, ME-Messsysteme GmbH, Henningsdorf, Germany) with 0.5N nominal force and an accuracy class of 0.5% to capture insertion forces. Signals were acquired using a measuring amplifier (GSV-4USB-SUB-D37, ME-Messsysteme GmbH) including an analog-digital converter (16 bit) and a sampling rate of 10 Hz. A light, 3D-printed adapter allowed for mounting the ST phantom onto the force sensor.

Electrodes were guided by a tube (inner diameter of 1.5mm) up to the approximate location of the round window of the phantom prior to insertion (Fig 2D). The goal of guiding the electrode prior to insertion was to avoid buckling, as this has been shown cause large variations in maximum forces despite of consistent insertion parameters [21]. Guide tube and force measurement setup were mechanically decoupled to avoid measurement falsifications. Relative positioning of the guide tube (and hence the electrode array) to the cochleostomy of the ST phantom was achieved by three micro manipulators.

### Insertion protocol

Three FLEX28 (MED-EL, Innsbruck, Austria) CI electrodes were used for testing. This specific electrode type was shown to yield the desired postoperative neural coverage in the majority of patients [38] and is hence the most commonly implanted electrode at Hannover Medical School (MHH) offered by this manufacturer [56]. Furthermore, its behavior during insertion has been studied by several other groups [21, 24, 26, 28, 30, 39], allowing for the comparison of the present results to the ones derived within these studies. The electrode was oriented with the orientation marker facing toward the modiolus (positive x direction, see Fig 2E). Position coordinates of the electrode were x = 0, y = 0, z = 0. To allow for the electrode to be guided before its insertion, it was inserted into the ST from electrode insertion depth EID_0_ = y0 = 0 mm to EID_4_ = y_start_ = 4 mm. Since the goal of the study was to measure insertion forces created by interaction of the electrode array and the intracochlear anatomy, the guide tube was placed in very close proximity to the cochleostomy (1 mm) to avoid the electrode touching the edge of the cochleostomy (Fig 2E).

Prior to insertion, the ST phantom was filled with a lubricant. As liquid soap is commonly used as a synthetic perilymph substitute for CI insertion testing [13, 17, 22, 25, 26], three different lubricant concentrations were tested in the following order: 90%, 50%, 10% liquid soap mixed with H_2_O. Each electrode was tested in a series of insertion cycles with constant lubrication and varying insertion speeds. Each insertion cycle consisted of a total of three insertions. Before each new cycle, the electrode location was zeroed at y_0_ and positioned to y_start_ manually. The order of the insertion cycles was as follows: one conditioning cycle with v_c_ = 0.5 mm/s, five speed cycles with v_s_ = 0.1, 0.25, 0.5, 1.0, 2.0 mm/s respectively and one repetition cycle with v_r_ = 0.5 mm/s. Each insertion had the following parameters: EID_max_ = y_max_ = 28 mm, pause at y_max_ of t = 2 s, retraction speed v_r_ = 0.5 mm/s, pause after retraction at y_start_ of t = 2 s. After a series of insertion cycles was finished, the electrode was soaked in H_2_0 for about 2h and then dried for over 2h. The ST phantom was washed prior to each change in lubrication as follows: 3 times flushing, then soaking for 16 h, both with H_2_O.

### Data evaluation

Data evaluation was performed in Matlab (version R2018a, The MathWorks Inc., USA). Several different factors were investigated following previously published studies on cochlear implant insertion forces [7, 22], including the force F_max_ at EID_max_, insertion Work W, as well as the snap of the insertion force profile F(y). Note that the latter was computed differently than the “jerk” value proposed by Nguyen et al., which was described by the root mean square (RMS) of the first derivative dF/dt of an insertion [7]. This metric is not suitable for the present study as the first time derivative is affected by insertion speed and leads to an overinterpretation of faster insertions (see S2 Appendix for clarification). Instead, the RMS of the speed independent snap is evaluated according to:

$$Snap=RMS(d^{2}F(y)/dt^{2})$$ (1)

Finally, the insertion work W was computed to yield overall expressions for how much “effort” is necessary to insert an electrode array. The insertion work describes the area under the force profile F(y) and is calculated by

W=∫F(y)dy. (2)

We decided to calculate the insertion work rather than the time dependent “total change in momentum” proposed by Nguyen et al. [7] in order to be able to compare different insertion speeds.

Statistical analysis was performed in Matlab as well using the Mann-Whitney U-test with 5% significance level.

## Results

### Electrode behavior over time–electrode conditioning

**Fig 3:**
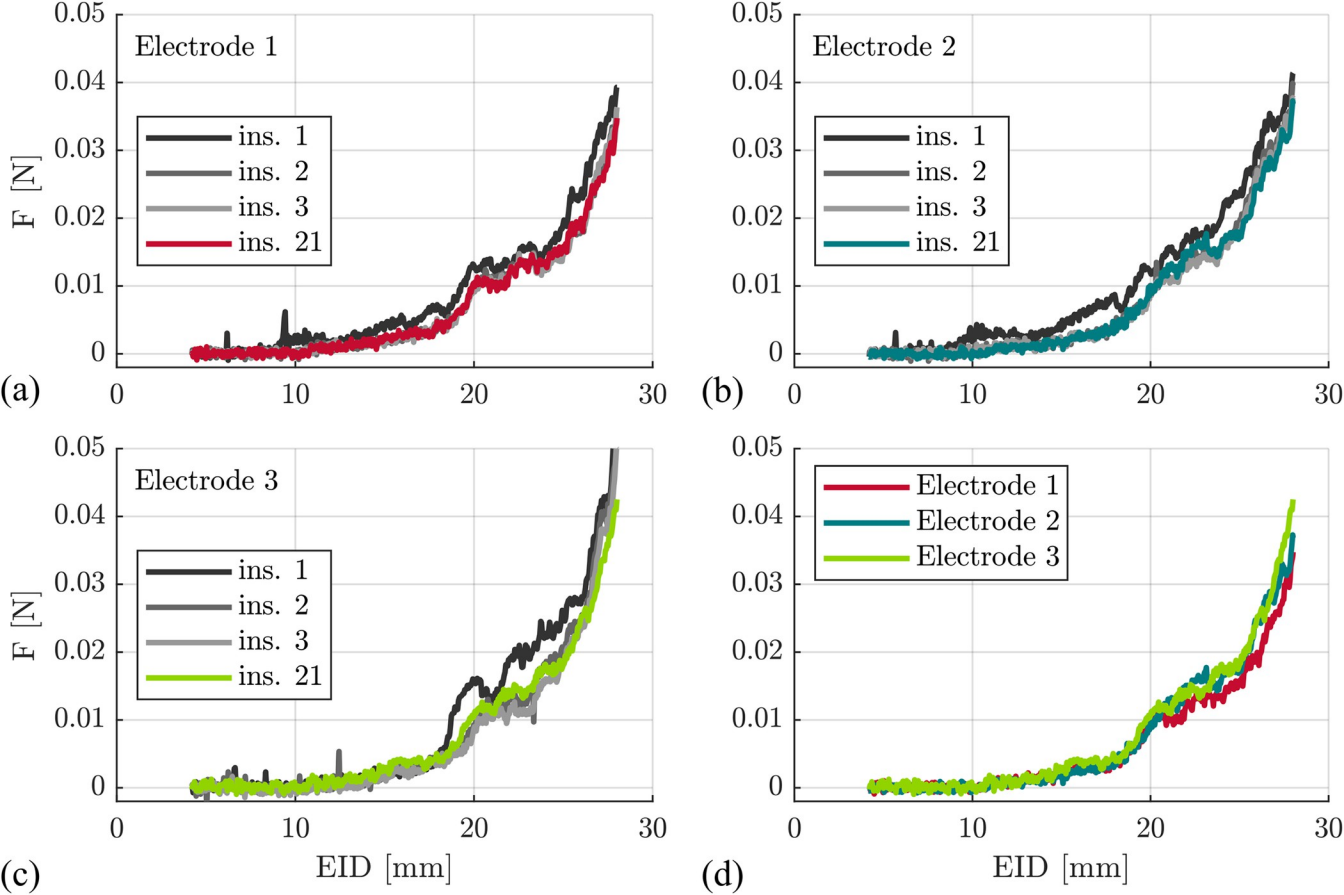
Insertion force profile of the three FLEX28 electrodes. Three electrodes in (a)-(c); v = 0.5 mm/s, lubrication 90% soap solution. Qualitatively only the very first insertion of each electrode appears to differ from the subsequent and last (ins. 21) insertion. Differences between the electrodes appear to become negligible after the conditioning cycle (d).

The three FLEX28 electrodes were inserted according to the protocol described above. In order to evaluate the comparability of insertions with different electrodes throughout the overall study, the very first insertion of the conditioning cycle of each electrode was compared with the second, third and last (21st) insertion of the respective electrode (v = 0.5 mm/s, lubrication 90%). The corresponding results are displayed in Fig 3A–3C and show that qualitatively, only the first insertion appears to differ from all others. Furthermore, differences between electrodes seem to become negligible after the conditioning cycle (Fig 3D). Quantitative comparisons were conducted using the work W of the individual insertions and confirmed only minimal changes in between electrodes and insertions after the first insertion of each electrode (Table 2), showing a 28% higher insertion work for the first insertion in comparison to the following ones.

**Table 2:** Insertion work for different electrodes. Insertion work W for the first insertion (Ins. 1) of each one of the three electrodes El. 1 to El. 3 is 28% higher than all subsequent ones (Ins. 2, 3 and 21). Only minor changes in work occur afterwards.

	W [Nm]			
	El. 1	El. 2	El. 3	mean
**Ins. 1**	0.21Nm	0.24Nm	0.24Nm	0.23Nm
**Ins. 2**	0.16Nm	0.18Nm	0.19Nm	0.18Nm
**Ins. 3**	0.16Nm	0.17Nm	0.17Nm	0.17Nm
**Ins. 21**	0.16Nm	0.18Nm	0.19Nm	0.18Nm

### Insertion force dependency on insertion speed and lubrication

**Fig 4:**
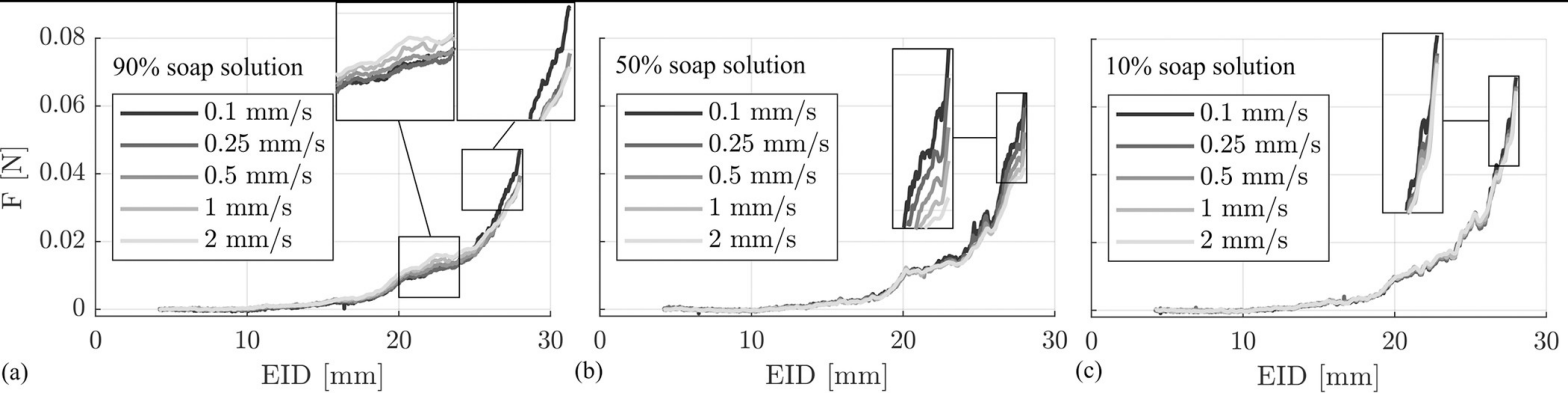
Mean electrode insertion forces. Forces averaged over all electrodes and each of the three insertions per cycle (n = 9 insertions). Maximum forces differ by insertion speed and concentration of the lubricant. Note that a snap in the force profile can be observed in all conditions.

The subsequent analysis was conducted to investigate the influence of insertion speed for the different lubrications. First, the insertion cycles of the three different electrodes were averaged for identical lubrications and insertion speeds, yielding the mean profile of 3 cycles (and hence 9 insertions) per speed and lubrication. The results, grouped by lubrication, are shown in Fig 4. Mean forces at particular locations (e.g. F_max_ at the end of each insertion) are stated in Table 3. For the highest concentration (90%), the force-EID curve shows qualitative differences especially at the region of EID = 23 mm (Fig 4A) where the largest insertion speed v = 2 mm/s yields the highest force F_max_ = 0.016 (±0.002) N. However, at the end of the insertion the force-EID profile is similar between speeds v = 0.25, 0.5, 1 and 2 mm/s. Mean max. forces for those speeds differ by 5% or less. Only the slowest insertion speed with F_max_ = 0.046 (±0.007) N shows a steeper slope, which was also confirmed by the statistical analysis which revealed significant differences only between 0.1 mm/s and the two fastest insertion speeds (1 mm/s and 2 mm/s respectively, see supplementary material).

**Table 3:** Mean maximum insertion forces by speed and lubrication. Maximal forces increase with decreasing soap solution of lubricant and decreasing insertion velocity. N = 9 insertions.

		F_max_ [N]			
		90%		50%	10%
		23mm	28mm	28mm	28mm
**v [mm/s]**	**0.1**	0.013+/-0.001	0.046+/-0.007	0.064+/-0.003	0.069+/-0.017
	**0.25**	0.012+/-0.001	0.039+/-0.005	0.057+/-0.002	0.066+/-0.017
	**0.5**	0.013+/-0.002	0.039+/-0.004	0.052+/-0.003	0.066+/-0.016
	**1**	0.015+/-0.002	0.037+/-0.004	0.047+/-0.001	0.065+/-0.016
	**2**	0.016+/-0.002	0.037+/-0.002	0.042+/-0.005	0.065+/-0.016

For the 50% lubrication, differences of the force-EID curves can be observed especially toward the end of the insertion where higher insertion speeds result yield lower insertion forces (Fig 4B). Statistical analysis revealed that each increase in insertion speed results in a significant reduction of maximum insertion forces (see S3 Appendix. Statistical Analysis. Table 2). The qualitative shape of the curve remains very similar.

For the most diluted lubrication of 10% there is hardly any qualitative difference in the mean force profile for the different insertion speeds (Fig 4C), which was confirmed by statistical analysis which revealed no significant differences. The largest difference in max. force F_max_ is no larger than 6%.

### Snap during electrode array insertion

**Fig 5:**
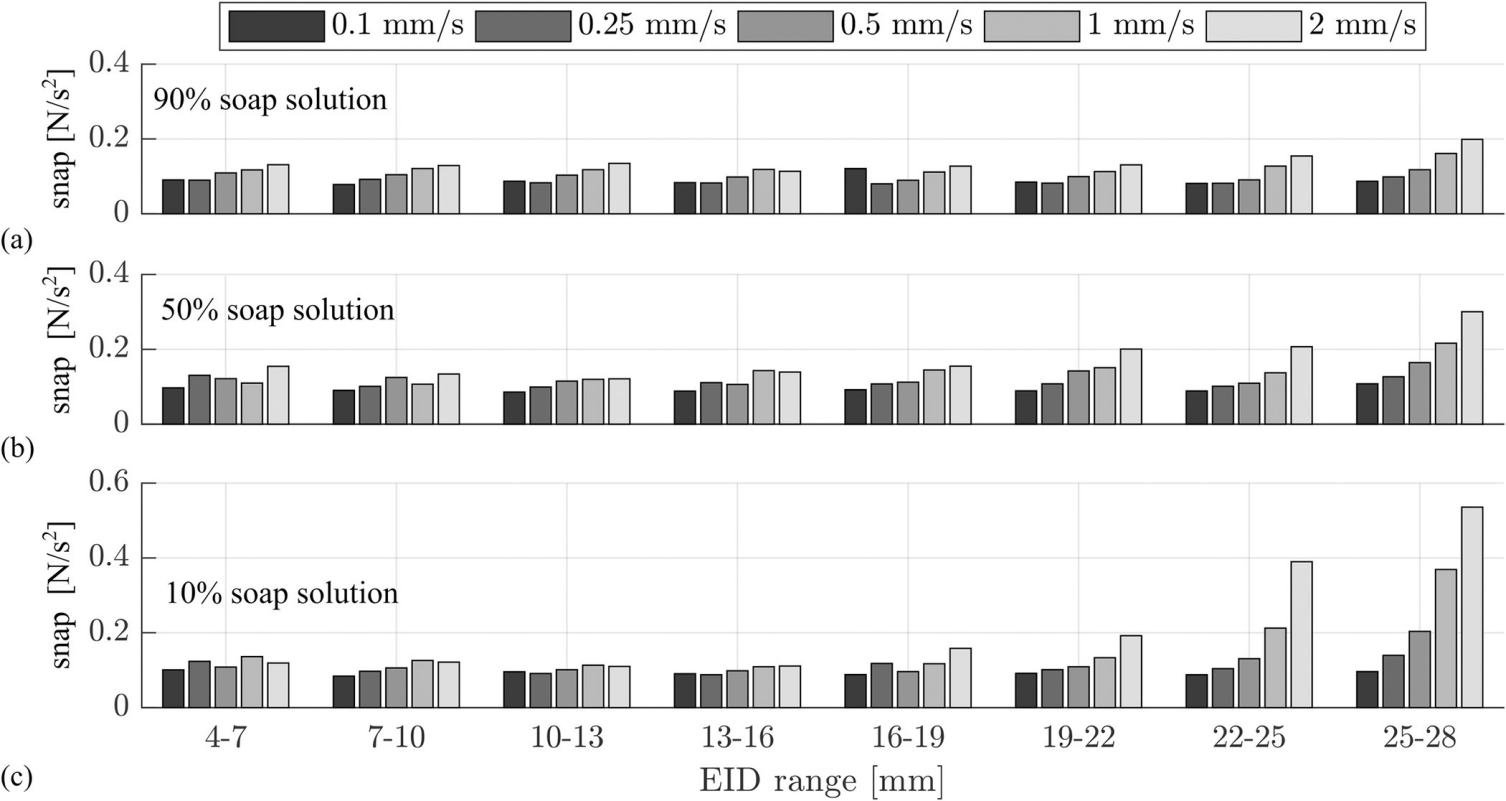
Snap of force profile. Snap is quantified by (1). Lower insertion speeds result in smoother force profiles and smaller snap. Values for larger insertion speeds v = 1 mm/s and v = 2 mm/s increase towards the end of insertion. The effect is more pronounced the lower the concentration of the lubricant. At 25–28 mm, the RMS of the snap increases significantly with increasing speed for all neighboring values (see S3 Appendix. Statistical Analysis. Table 9).

As described before, the snap of the insertion force profile was investigated as well. Fig 5 depicts the RMS of the second time derivate of the force at different ranges of EID where a low RMS value corresponds to low snap and hence a section with a smooth insertion force profile. In general, lower insertion speeds result in smaller snap, i.e., in smoother force profiles. Snap values for slow insertion speeds remain constant over the whole insertion process. However, beyond an EID of 19 mm the values for v = 1 mm/s and v = 2 mm/s increase in all lubrications. This effect is most prominent for the 10% lubrication and largest insertion speed v = 2 mm/s: here, the snap at 25-28mm is more than 3 times as high as the one for 16-19mm (Fig 5C), and more than 2.5 times as high as the value for 25–28 mm with the 90% lubrication (Fig 5A). This observation is confirmed by the Mann-Whitney U-test as the RMS of the snap from 25–28 mm increases significantly for all adjacent insertion speeds (see S3 Appendix. Statistical Analysis. Table 9). In contrast thereto, significant differences in RMS values for the least diluted lubrication 90% could only be observed from 0.5 to 1 and 1 to 2 mm/s (p = 0.003 and p = 0.031 respectively).

**Fig 6:**
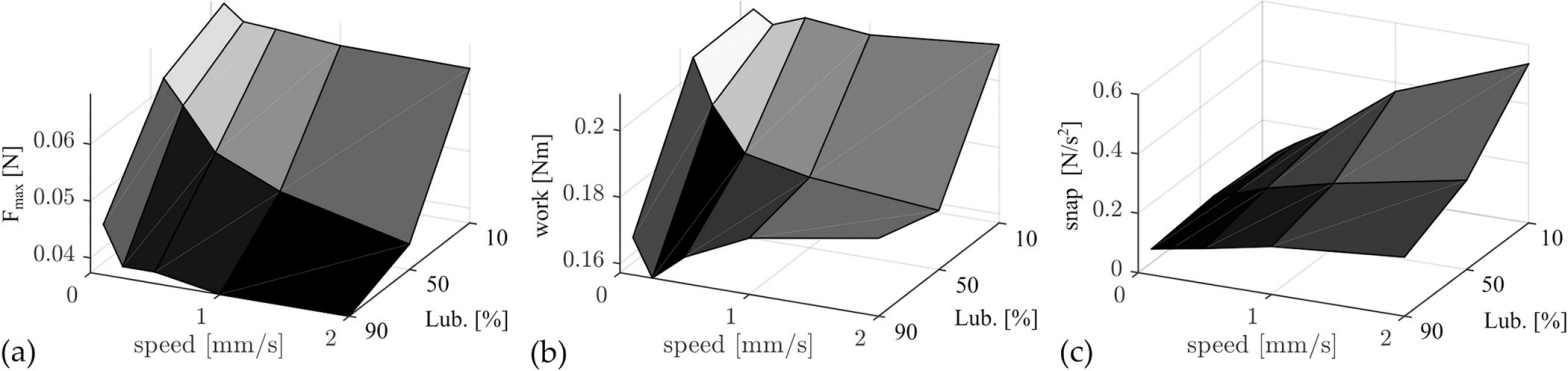
Results for influence of insertion speed and lubrication onto insertion behavior. (a) for higher lubrications of 50% and 90% soap solution F_max_ decreases with increasing insertion velocity. (b) Insertion work W shows almost constant values (10%), decreasing work (50%), and increasing work (90%) for rising insertion speeds depending on the concentration of the lubricant. (c) Snap, representatively only shown for 25–28 mm EID, is more pronounced for the lower concentrated lubrication and large insertion speeds.

Fig 6 summarizes the results above within three surface plots describing the influence of insertion speed and lubrication onto (a) the maximal insertion force, (b) the work necessary for inserting the electrode and (c) the smoothness of the insertion force over time (i.e. snap). Regarding the maximal force, the most drastic changes can be observed for the different lubrications where the least concentration (10% soap solution) yields the highest insertion forces for all velocities (Fig 6A). The latter further shows that for the employed insertion test setup, higher velocities result in smaller force values. However, force changes for different velocities are not very pronounced for the 90% or the 10% lubrications. The mean insertion work (n = 9 insertions) necessary for a whole insertion is shown in Fig 6B. For the least concentrated lubrication (10% soap solution) insertion work is the largest with almost no differences between the different insertion speeds. As mentioned before, no statistical difference in maximum force could be observed for the different insertion speeds (see S3 Appendix. Statistical Analysis. Table 6). For the 50% lubrication a decrease in work for increasing insertion speeds can be observed. This effect is most pronounced for high insertion speeds as work for the faster speeds from 0.5 mm/s and beyond is significantly decreased compared to the slowest insertion speed (p < 0.001, see S3 Appendix. Statistical Analysis. Table 5). Interestingly, the insertion work shows the opposite effect for the 90% lubrication where overall, work significantly increases for higher velocities (v = 2 mm/s is significantly larger than 0.25 and 0.5 mm/s, p = 0.0188 and p = 0.04 respectively). Only between speeds v = 0.1 mm/s and v = 0.25 mm/s there is a 7% dip. The summary on the snap investigation (Fig 6C) shows values at 25–28 mm EID representatively: overall, insertion force profiles are smoother for high viscosities and low velocities.

## Discussion

The impact of CI electrode array insertion on the cochlear anatomy has interested research groups since the early days of CI implantation. New surgical assisting systems protrude into the operating room (OR) [6–8], which allow for precise control of insertion parameters (e.g. insertion trajectory, insertion speed) beyond the possibilities even the very skilled hand of an experienced surgeon can achieve. Hence, experimental investigations on electrode insertion behavior gain new interest. Unfortunately, differences in test setups and ST phantom designs create large variations and even contradicting results in between research groups. Standardization of the testing methods and better insights into the underlying mechanical phenomena leading to those findings is therefore of key importance. The results presented in this study were generated to understand influences of the test setups and their influence onto experimental findings, especially regarding the interdependence of insertion forces, insertion speed, and lubrication.

The behavior and interaction of the system components (I) *electrode*, (II) *ST phantom*, and (III) *lubricant* are directly influenced by rigid boundary conditions caused by the *test setup* (i) and ST insertion phantom *surface area* (ii). Both directly influence the *dynamic viscoelastic behavior* (iii) of the electrode and the *dynamic mechanical interaction* (iv) of all system components.

The presented *testing setup* (i) and insertion protocol allows for a reliable repeatability of electrode insertion testing with consistent electrode behavior and minimal changes of insertion force after the conditioning cycle (see Table 2). The small standard deviations in F_max_ at v = 0.5 mm/s with 4.0 mN for the 90% lubrication and 3.0 mN for 50%, respectively (Table 3), are in line with results from other groups [17, 26]. This good repeatability is most likely owed to two main effects: the first one is the guidance of the electrode array prior to entering the ST phantom. By using a guide tube, the viscoelastic behavior (iii) of the system component electrode is reduced to the intracochlear part of the array and does not portray any extracochlear bending behavior. Furthermore, contact to the edge of the cochleostomy is prevented, and only intracochlear forces are measured due to the decoupling of guide tube and force sensor setup. Hence, friction forces from the electrode lead occurring within the guide tube do not contribute to the force profile. Both Kobler et al. and Aebischer et al. used the same procedure and achieved the same repeatability. This aspect is thus very crucial. With a more manual surgery-oriented test setup and by not constraining extracochlear buckling, Zuniga et al. described that complete insertions occurred for very slow speeds, but did not occur in > 50% of the cases for speeds ≥ 0.9 mm/s [21]. Additionally, Dohr et al. [25] describe a kinking of the electrode lead, leading to a drop in force and a falsification of the subsequent insertion force recordings with their setup. Within the present study, no kinking could be observed for any of the 153 performed insertions, most likely due to the electrode array guidance prior to insertion. Consequently, it is advisable to guide the electrode accordingly when investigating the interaction of electrode array and intracochlear structures. This suppresses largely any dynamic bending behavior in this part and leads to consistent and repeatable results.

The second characteristic yielding a high repeatability is the mechanical conditioning of the electrode. The CI electrode is a complex silicone rubber composite, comprised of platinum alloy contacts, platinum alloy wave shaped wires and a silicone rubber matrix. During the first insertion and bending of the electrode, the wires are plastically deformed, physical bonds in between platinum parts and silicone matrix are broken, and form closure of compound partners are reorganized. This leads to a change in mechanical properties and strain softening during in the first insertions, which is known as the Mullins’ effect and typical for all rubber composites [41]. Mullins describes the effect to be largest between the first and second cycle, which can also be observed within our data (see Fig 2). Literature also describes the stress softening after the conditioning phase to stay constant for silicone rubber composites as long as deformation parameters (e.g. ST geometry) do not change [57]. Zuniga et al. report similar observations in CI insertion experiments [21]. Consequently, when aiming at performing insertion experiments and comparing results with different insertion parameters, we suggest a test setup with force sensors beneath the phantom, electrode guidance prior to ST phantom entry and conditioning electrode arrays to focus on the *viscoelastic behavior* (iii) of the *electrode* within the intracochlear part. However, it must also be noted that those settings are clearly a deviation from current manual and assisted electrode insertion techniques in the OR [7, 24], i.e. results must be interpreted within the boundary conditions of the experiment. Nevertheless, future developments especially in minimally invasive cochlear implantation [6, 8] have the chance to transfer a well-defined and standardized insertion setup into the surgical world and patient treatment.

The ST phantom *surface area* (ii) was lubricated to largely suppresses material dependent adhesion forces [58, 59] and to focus only onto the geometrically driven contact area. This approach seems to be valid as F_max_ values measured within this study (see Table 3) are well within the forces measured by Aebischer et al. and their friction optimized hydrophilic insertion phantom [17, 22]. The presented ST insertion phantom is an anatomically correct representation of the mean anatomy of the human ST. As the human cochlea does not only vary in size [3] but also orientation of cochlear cross-sections in between individuals [32, 34], creating such a mean model is not trivial. To compile the mean model used in this study, the methods and data described in [51] have been used, preserving the common anatomical features of the cross-sectional geometry without falsification (see Fig 1). This is highly relevant, as geometry has a direct impact onto the contact area between the electrode array and the lateral wall [35], thus effecting friction forces. Schurzig et al. have shown that single anatomical parameters of the ST (e.g., ST height and spiral length) do not necessarily correlate [34]. Hence, also large cochleae can have a small ST height and vice versa. Consequently, studying the impact of single insertion parameters (e.g., speed, trajectory) requires an anatomically correct mean model, being independent of individual characteristics.

The anatomical parameters for the insertion phantom (Table 1) are within the range of values reported in literature [38, 49, 55]. As the data for the ST phantom is not measured at the level of the bone, A_ST_ and B_ST_ are expected to be slightly smaller as corresponding values from clinical scans [35]. H_ST_ and H_S, ST_ as well as CDL_LW, ST_ and CDL_ST_ are also expected to be smaller as the phantom only represents the ST prior to the helicotrema [60]. The presented ST phantom also comprises a pressure release hole at the apical end of the model. As friction forces and fluid pressure build up both contribute to insertion forces to some degree, the motivation for such a hole was to largely reduce pressure increase which is caused by volume displacement of inserting the electrode into the lubricant (e.g. perilymph) [42, 43]. It is yet unclear to which degree both phenomena contribute to the overall mechanical interaction (iv) of the system components, and they cannot be separated in the measured bulk force profile.

**Fig 7:**
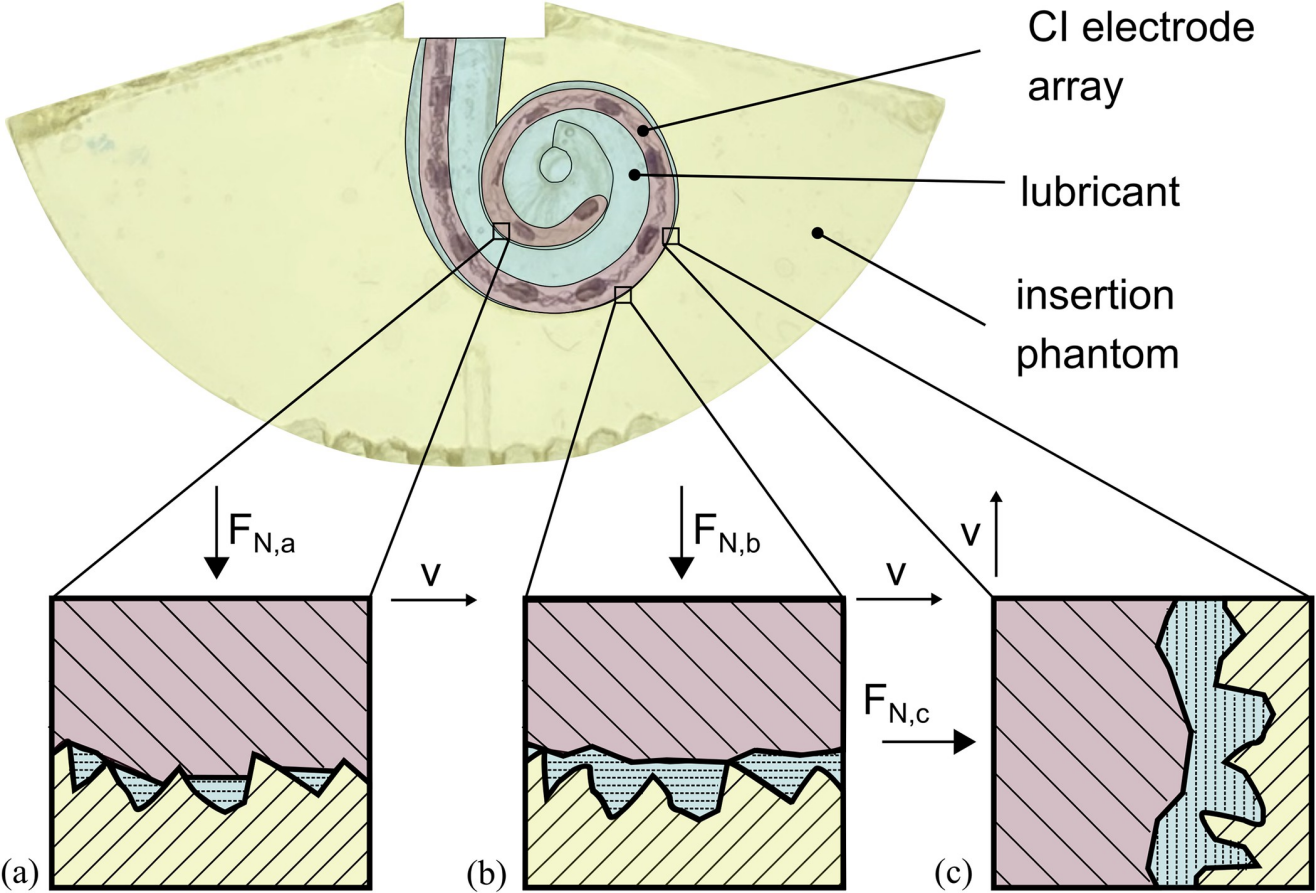
Different states of friction during CI electrode array insertion. (a) In boundary friction the lubricating film is collapsed or only of molecular thickness. (b) In mixed friction the surface asperities of the array and ST insertion phantom are indirect contact. (c) In hydrodynamic friction array and ST phantom surface are completely separated by the lubricant. Due to different local normal forces F_N_ *[22]*, different frictional states can occur along the array simultaneously *[47]*. The effect is further driven by insertion speed and concentration of the lubricant.

By suppressing pressure increase, friction forces can hence be assumed to majorly contribute to the mechanical interaction (iv) in our system and to be the main driver for the electrode insertion forces presented. Stribeck showed that in lubricated settings, different states of friction can occur [47], and research on rubber friction [45, 59, 61] suggest that they can directly be attributed to CI electrode insertion: in *boundary* lubrication, the lubricant is either collapsed or only of molecular thickness and both frictional partners can be in direct contact (Fig 7A). In *mixed* lubrication, electrode and ST surface are not completely separated by the lubricant and surface asperities of both frictional partners can be in direct contact (Fig 7B). In *hydrodynamic* friction, CI electrode and ST surface area are completely separated by the lubricant (Fig 7C). In his studies, Stribeck pointed out that due to different operating and local loading conditions, these frictional states usually superimpose and are difficult to separate. Furthermore, the proportion of contribution to the overall forces varies in between loading conditions. We assume the same to be valid for CI insertion measurements, as the outer geometry of the flexible electrode array changes along its length and the geometry of the lateral wall changes along the cochlear spiral [32, 34]. Hence, the local contact area and normal force F_N_ between both frictional partners is constantly changing during the course of an insertion [22, 35].

Our data suggests that boundary lubrication with predominant direct contact between electrode and phantom is the main cause of the nearly speed independent results for F_max_ and W for the 10% lubrication (Fig 6A and 6B): this lubrication is the least viscous one, and there are almost no qualitative differences in the force profiles (Fig 4C). Due to the low concentration, it can occur that the thin liquid film between electrode and phantom collapses [45], thus yielding direct contact. Complementarily, Arvanitaki et al. describe friction to be independent of velocity in their experiments with silicone rubber and low viscosity lubricants in the boundary regime [59]. Another characteristic supporting this assumption is the jerking motion which can be quantified by the snap according to (1). Our assumption is, that this metric portrays different phenomena than a common stick-slip characteristic. Stick-slip is driven by adhesion between two rubbing surfaces and can be characterized by a periodic oscillation between static and kinetic friction force [62]. However, as shown in Fig 5 snap increases with EID and insertion speed, which can qualitatively be observed in all recorded force profiles (e.g., Fig 4, magnified regions). In contrast stick-slip would decrease with increasing velocity [62, 63]. Consequently, we hypothesize that this metric quantifies some locking in direct contact between parts of the electrode and phantom surfaces. As Fig 5 quantifies this local phenomenon it can be noted that values for the 10% lubrication are most pronounced. A larger snap for poorer lubricated system further underlines our theory This snap-speed dependency has been observed for CI electrode insertion before [21]. Furthermore, the largest standard deviation in F_max_ for this lubrication (Table 2) is most likely a direct effect of the snapping motions. As those become larger near the end of the insertion, they lead to an inhomogeneous variation of F_max_—an observation also described by Kobler et al. [26]. Speed independent insertion forces as described above are also reported by Avci et al. [13] in their electrode insertion experiments with an acrylic insertion phantom (insertion speeds between 0.05 and 2 mm/s; 10% soap solution lubrication).

The speed dependency of insertion forces, with decreasing force and work values for increasing insertion velocities indicates that mixed friction (Fig 7B) is most likely the predominant state in the 50% lubrication. In theory, when a block slides over a surface with the velocity v, a wedge-shaped film of liquid builds up between the two surfaces and exerts a pressure which tends to separate the two bodies. This behavior forms the basis of bearing lubrication [47] and was studied in more detail for rubber in the skid behavior of tires by Grosch and Schallamach [45, 46]. The minimum liquid thickness h is given by:

$$h=constant\sqrt{a^{2}b\eta v/F_{N}}$$ (3)

with a and b being block surface area dimensions, η being the liquid viscosity, v the sliding velocity, and F_N_ the normal force [64]. Eq (3) explains the negative velocity dependency of the force profiles of the 50% lubrication (Fig 6A). Compared to the boundary lubrication state, larger velocities lead to better lubrication and growing separation of electrode and insertion phantom as the film thickness increases [45]. Hence, a lower force is needed for the overall insertion. The present but reduced snap characteristics (Fig 5B) are an additional indicator for mixed friction. A decrease in maximal insertion forces with increasing speed was also observed by other groups when inserting into acrylic phantoms [24, 28], and Zhang et al. argue that lubrication effects are responsible for their findings, which is in line with our hypothesis.

The insertion work results W (Fig 6B) show an inverse behavior for 90% lubrication compared to 50%. This positive speed dependency of insertion forces, but yet smaller overall forces as compared to 10% lubrication, indicates hydrodynamic friction [47, 59]. The increased concentration of the lubricant increases its viscosity and enhances wettability. Hence, separation of both frictional partners as defined in Eq (3) occurs, reducing overall forces. However, internal shear processes within the lubricant lead to an increase in insertion force with increasing speed. The smallest snap values (Fig 5) compared to all other lubrications further support the theory of a reduced real contact area, which would lead to larger hydrodynamic friction.

It remains questionable to which degree hydrodynamic friction contributes to the results of other research groups who observed a similar positive force-speed dependency [21–23, 29, 31, 39] When lubricating only with water or saline solution [21, 23, 29], the wettability is poor and the lubrication film can collapse [45], leading to an almost dry contact area which would also show a similar positive force-speed relationship [58, 65]. This effect explains the observations by Starovoyt et al. who describe that they were only able to insert half of the electrode array when using saline solution in 3D printed ST models in contrast to deeper insertions when using soap solution [19]. Furthermore, when lubricating with absorbing fluids like alcohols (e.g. glycerin solution [12, 27, 28, 44]), increased friction forces can occur as the silicone rubber of the electrode array or polymeric phantom surface might be subjected to softening and swelling [66]. Unfortunately, some groups do not specify their lubricant composition when studying frictional behavior [24, 25, 48]. Fluid pressure is also known to lead to larger forces with rising speeds and has been described for CI insertion and lower viscous lubricants [42, 43]. The groups above reporting a positive force-speed dependency only rely on a cochleostomy as pressure release. Hence, rising forces would be in line with findings from Todt et al., who show that the size of cochlear opening significantly effects pressure forces, with smaller openings showing larger pressures [67]. However, when calculating the force on the electrode array by transferring the reported static insertion pressures to the diameter of the inserted array, those forces are presumably by at least an order of magnitude smaller than the measured friction forces [42, 43] and should be well below the reported standard deviation in Table 3. To add to the complex mechanical interaction within the ST, the electrode itself contributes to non-linear, time-dependent behavior. For some of the groups mentioned, the electrode is designed of straight wires [17, 22, 29, 31]. This difference in design is directly attributed to a difference in viscoelastic bending and increased normal force F_N_ [22, 44], which again is directly attributed to a reduction of lubricant film thickness as per (3) and an increase in friction force [47]. Furthermore, not guiding the electrode also adds the extracochlear part to contribute to the overall bulk forces as discussed above. This observation is likely to be even more pronounced for wave-shaped wired electrodes [21, 23, 25, 29, 39] and a spring-like buckling behavior could also be attributed to a positive force-speed relationship.

When using the insertion phantom, testing setup, and insertion protocol described above, the results obtained cannot be transferred to the surgical world directly. The special characteristics of this lab setup are an idealization of the complex interactions during CI implantation and only an abstraction. A transfer of measured forces to possible implications for tissue damage or electrode insertion trauma [11] must be considered very carefully. Human tissue also shows viscoelastic behavior [20]. Consequently, the results obtained from testing rupture behavior of cochlear tissues [68, 69] are directly attributed to the parameters of the test setup (e.g. testing speed and probe diameter) and are difficult to compare to discrete force results obtained with rigid ST phantoms. It has been described that individual anatomical features have an impact on insertion forces [13, 14, 17]. Hence, each CI patient would most likely show different characteristics in the insertion force profile. The pressure release hole in the apex of the ST phantom is anatomically incorrect and was designed purely for the benefits described above. Furthermore, the viscosity of perilymph is most likely more watery and less viscous than the 90% soap solution used in this study [70].

The discussion above shows that multiple mechanical phenomena can act simultaneously during insertion, and the single components of the dynamic system can show time-dependent behavior. Consequently, when aiming at studying underlying mechanisms responsible for insertion forces (e.g. insertion speed, insertion angle, cochlear geometry), the single phenomena need to be decoupled by the boundary conditions of the test setup as they superimpose in the measured bulk force profile. The degree to which electrode insertion forces during CI surgery are influenced by the viscoelastic behavior of a specific electrode type, the friction behavior within the ST, or fluid mechanical forces also remains to be analyzed in future research.

## Conclusion

An anatomically correct and freely available physical insertion phantom based on an average representation of the human ST was developed within this study. A test setup and protocol are presented which allow for standardized, repeatable electrode insertion testing with small force variations under equal test conditions. The presented data shows a clear dependency of measured bulk forces on electrode conditioning, electrode insertion speed, and lubrication. It could be shown that certain phenomena known from lubricated rubber friction are likely responsible for these dependencies, and it is shown that the specific dependency of speed and insertion forces is strongly dependent on the boundary conditions of the test setup. This emphasizes the necessity for a clear definition of the test environment as the latter has a strong influence on the results obtained. Further research is necessary for transferring experimental findings onto patient treatment and deriving resulting implications for local forces on human tissue.

## Supporting information

S1 Appendix Scala tympani mean insertion phantom. Transformation from the cochlear coordinate system to newly defined insertion coordinate system.
(DOCX)

S2 Appendix Data evaluation snap. Example to demonstrate that calculation of snap is independent from insertion speed in contrast to value for jerk.
(DOCX)

S3 Appendix Results statistical analysis. Results of the statistical analysis for maximum insertion force, work, and snap.
(DOCX)

S1 Data Insertion testing data. Data of CI electrode array insertion testing.
(ZIP)
