# Supplementary material for: On the interdependence of insertion forces, insertion speed, and lubrication: Aspects to consider when testing cochlear implant electrodes
Source: PLoS One. 2024 Jan 24;19(1):e0295121. doi: 10.1371/journal.pone.0295121 (PMC10807833; doi:10.1371/journal.pone.0295121)
Supplement: S1 Appendix — Transformation from the cochlear coordinate system to newly defined insertion coordinate system. (DOCX) [file pone.0295121.s001.docx]

S1 Appendix. Scala tympani mean insertion phantom. Supplemental material to manuscript: On the interdependence of insertion forces, insertion speed, and lubrication: aspects to consider when testing cochlear implant electrodes

Max Fröhlich^1,2,*^, Daniel Schurzig^1,2^, Thomas S. Rau^2,3^, Thomas Lenarz^2,3^

**Methodology**

**Scala Tympani Mean Insertion Phantom**

The transformation from the cochlear coordinate system (CCS) to the newly defined insertion coordinate system (ICS) for the ST mean model can be described by the following (see Fig. 8):

- Translation vector: $v= \left( \begin{matrix} 4.64 \\ 0.7 \\ 0 \end{matrix} \right)$
- Rotation R_z_ by$\alpha=63,98^{\circ}$


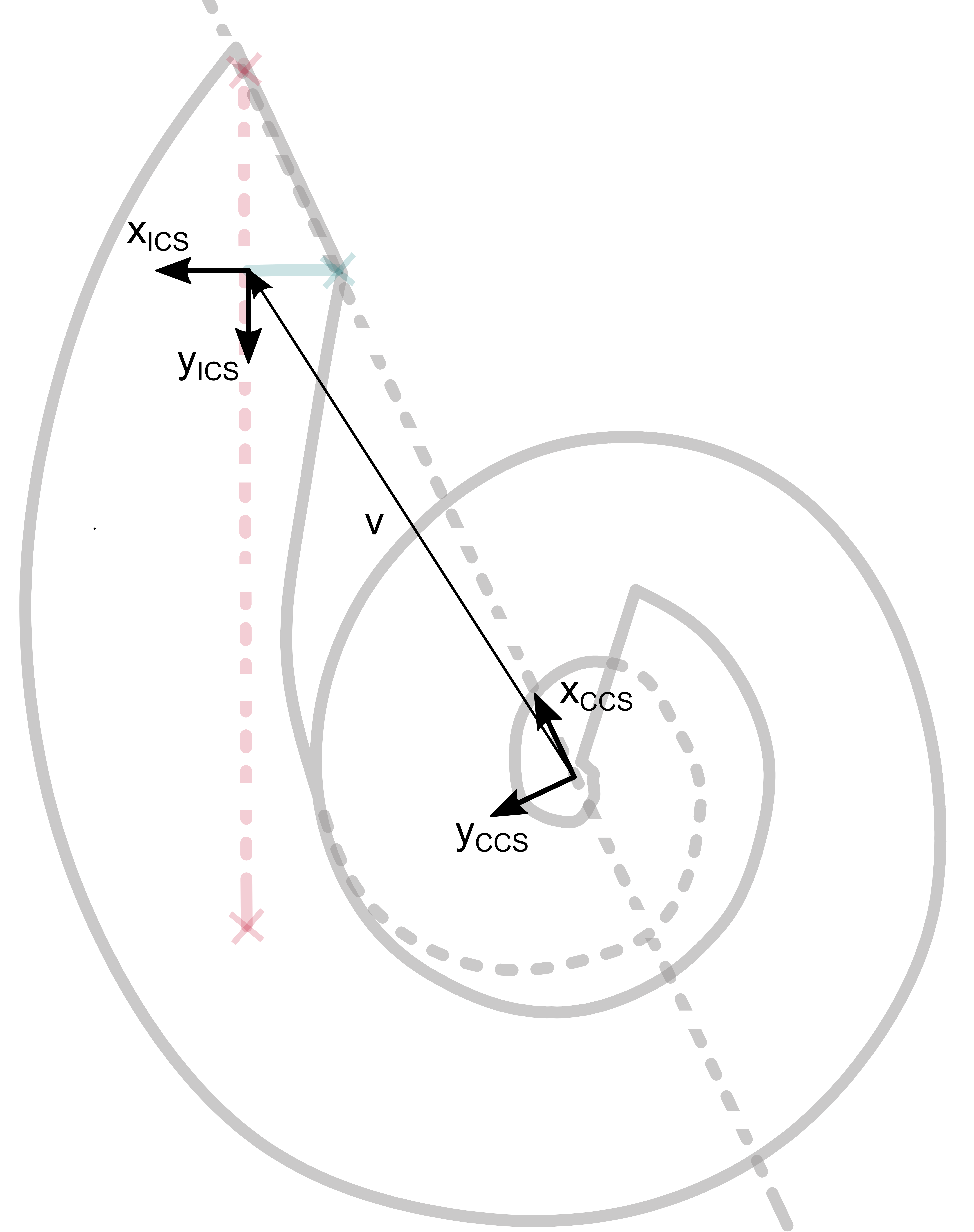


**Fig. 8.** **Transformation of ST mean insertion phantom.** Transformation of the cochlear coordinate system (CCS) to the insertion coordinate system (ICS) of the ST mean model. The CCS is translated by the vector v and a rotation R_z_.
