# Supplementary material for: On the interdependence of insertion forces, insertion speed, and lubrication: Aspects to consider when testing cochlear implant electrodes
Source: PLoS One. 2024 Jan 24;19(1):e0295121. doi: 10.1371/journal.pone.0295121 (PMC10807833; doi:10.1371/journal.pone.0295121)
Supplement: S2 Appendix — Example to demonstrate that calculation of snap is independent from insertion speed in contrast to value for jerk. (DOCX) [file pone.0295121.s002.docx]

S2 Appendix. Data evaluation snap

Supplemental material to manuscript: On the interdependence of insertion forces, insertion speed, and lubrication: aspects to consider when testing cochlear implant electrodes

Max Fröhlich^1,2,*^, Daniel Schurzig^1,2^, Thomas S. Rau^2,3^, Thomas Lenarz^2,3^

**Methodology**

**Data Evaluation**

The snap was validated according to (1) and not with the proposed “jerk” value by Nguyen et al. [7]. To explain why we decided against the jerk value, the two artificial force profiles displayed in Fig. 9a were generated. The right column shows the RMS values of the signals displayed on the left. Both profiles represent 3rd order polynomial functions ending at a peak force of 100mN at t = 20s (v = 0.1mm/s) and t = 1s (v = 2mm/s) respectively. Force variations or noise signals with amplitudes 20mN and 10mN were added to the 0.1mm/s and 2mm/s profiles respectively. Hence, an analysis of the force profiles should yield that the 2mm/s insertion is smoother than the 0.1mm/s insertion. However, the RMS in Fig 9 shows that the ones of the first derivative (Fig. 9b) are predominantly defined by the different insertion speeds. The result is a higher value for the fast insertion although the applied noise signal had a smaller amplitude. The RMS of the second derivative d^2^F/dt^2^, however, yields the expected result of a higher RMS value for the noisier 0.1 mm/s signal (Fig. 9c).


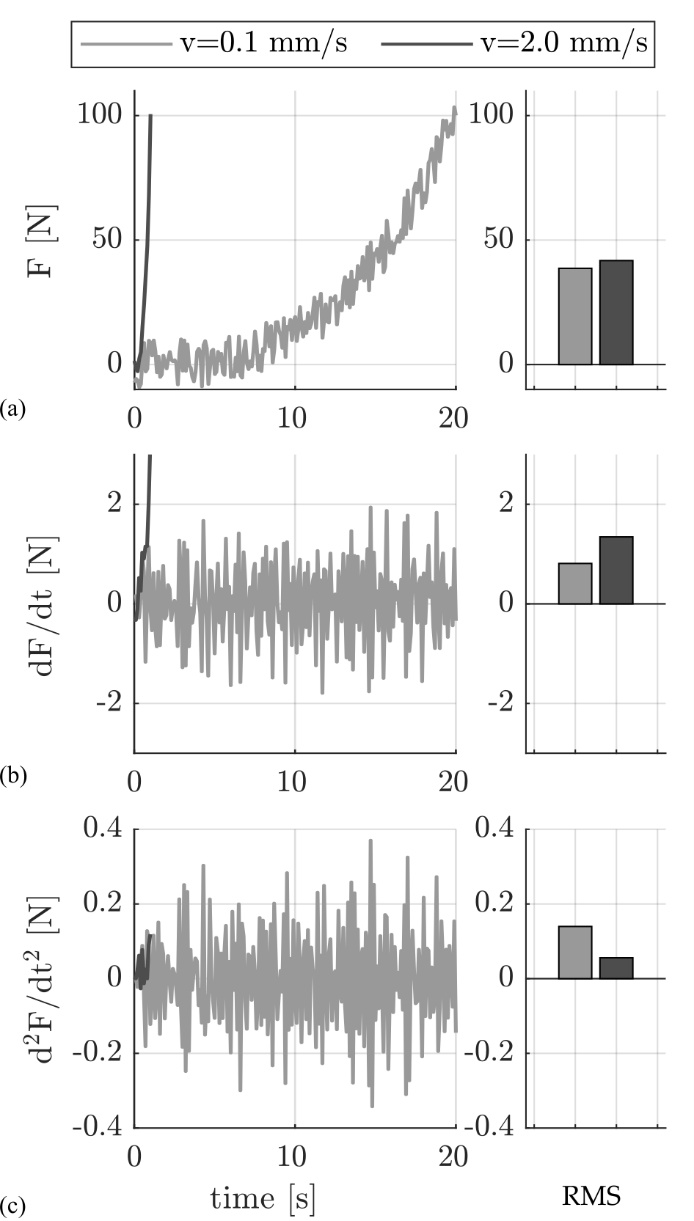


**Fig. 9.** (a) artificial insertion force profiles with a noise floor of the slow insertion (v = 0.1mm/s) which is twice as high as the one of the fast insertion (v = 2mm/s). (b) The first derivatives dF/dt (left column) and the corresponding RMS values (right column) showing a higher value for the lower noise floor of v=2mm/s. (c) The RMS value of the second derivative d^2^F/dt^2^ accurately displays the higher noise of the slow insertion.

| 7. | Nguyen Y, Kazmitcheff G, De Seta D, Miroir M, Ferrary E, Sterkers O. Definition of metrics to evaluate cochlear array insertion forces performed with forceps, insertion tool, or motorized tool in temporal bone specimens. BioMed research international. 2014; 2014: p. 532570. |
| --- | --- |

**References**
