# Supplementary material for: On the interdependence of insertion forces, insertion speed, and lubrication: Aspects to consider when testing cochlear implant electrodes
Source: PLoS One. 2024 Jan 24;19(1):e0295121. doi: 10.1371/journal.pone.0295121 (PMC10807833; doi:10.1371/journal.pone.0295121)
Supplement: S3 Appendix — Results of the statistical analysis for maximum insertion force, work, and snap. (DOCX) [file pone.0295121.s003.docx]

S3 Appendix. Results Statistical Analysis.

Supplemental material to manuscript: On the interdependence of insertion forces, insertion speed, and lubrication: aspects to consider when testing cochlear implant electrodes

Max Fröhlich^1,2,*^, Daniel Schurzig^1,2^, Thomas S. Rau^2,3^, Thomas Lenarz^2,3^

**Results**

**Statistical Analysis**

**Force F_max_**

**S3 Table 1. Results Statistical Analysis F_max_** Lubrication 90%. Resulting p-values from statistical analysis (Mann-Whitney U-test, 5% significance level).

|  | v [mm/s] | | | | | |
| --- | --- | --- | --- | --- | --- | --- |
| v [mm/s] |  | **0.1** | **0.25** | **0.5** | **1.0** | **2.0** |
|  | **0.1** |  | 0.050309 | 0.077005 | **0.002756** | **0.000041** |
|  | **0.25** | 0.050309 |  | 1.000000 | 0.666475 | 1.000000 |
|  | **0.5** | 0.077005 | 1.000000 |  | 0.222419 | 0.222419 |
|  | **1.0** | **0.002756** | 0.666475 | 0.222419 |  | 0.863307 |
|  | **2.0** | **0.000041** | 1.000000 | 0.222419 | 0.863307 |  |

**S3 Table 2. Results Statistical Analysis F_max_** Lubrication 50%. Resulting p-values from statistical analysis (Mann-Whitney U-test, 5% significance level).

|  | v [mm/s] | | | | | |
| --- | --- | --- | --- | --- | --- | --- |
| v [mm/s] |  | **0.1** | **0.25** | **0.5** | **1.0** | **2.0** |
|  | **0.1** |  | **0.003990** | **0.000041** | **0.000041** | **0.000041** |
|  | **0.25** | **0.003990** |  | **0.000041** | **0.000041** | **0.000041** |
|  | **0.5** | **0.000041** | **0.000041** |  | **0.000041** | **0.000082** |
|  | **1.0** | **0.000041** | **0.000041** | **0.000041** |  | **0.010613** |
|  | **2.0** | **0.000041** | **0.000041** | **0.000082** | **0.010613** |  |

**S3 Table 3. Results Statistical Analysis F_max_** Lubrication 10%. Resulting p-values from statistical analysis (Mann-Whitney U-test, 5% significance level).

|  | v [mm/s] | | | | | |
| --- | --- | --- | --- | --- | --- | --- |
| v [mm/s] |  | **0.1** | **0.25** | **0.5** | **1.0** | **2.0** |
|  | **0.1** |  | 0.077005 | 0.258083 | 0.062526 | 0.050309 |
|  | **0.25** | 0.077005 |  | 0.796174 | 0.161497 | 0.222419 |
|  | **0.5** | 0.258083 | 0.796174 |  | 0.604813 | 0.730440 |
|  | **1.0** | 0.062526 | 0.161497 | 0.604813 |  | 0.931427 |
|  | **2.0** | 0.050309 | 0.222419 | 0.730440 | 0.931427 |  |

**Work W_max_**

**S3 Table 4. Results Statistical Analysis W_max_** Lubrication 90%. Resulting p-values from statistical analysis (Mann-Whitney U-test, 5% significance level).

|  | v [mm/s] | | | | | |
| --- | --- | --- | --- | --- | --- | --- |
| v [mm/s] |  | **0.1** | **0.25** | **0.5** | **1.0** | **2.0** |
|  | **0.1** |  | 0.135911 | 0.604813 | 0.489428 | 0.135911 |
|  | **0.25** | 0.135911 |  | 0.297326 | 0.050309 | **0.018758** |
|  | **0.5** | 0.604813 | 0.297326 |  | 0.297326 | **0.039984** |
|  | **1.0** | 0.489428 | 0.050309 | 0.297326 |  | 0.190251 |
|  | **2.0** | 0.135911 | **0.018758** | **0.039984** | 0.190251 |  |

**S3 Table 5. Results Statistical Analysis W_max_** Lubrication 50%. Resulting p-values from statistical analysis (Mann-Whitney U-test, 5% significance level).

|  | v [mm/s] | | | | | |
| --- | --- | --- | --- | --- | --- | --- |
| v [mm/s] |  | **0.1** | **0.25** | **0.5** | **1.0** | **2.0** |
|  | **0.1** |  | 0.190251 | **0.007775** | **0.001851** | **0.001234** |
|  | **0.25** | 0.190251 |  | 0.258083 | 0.077005 | 0.077005 |
|  | **0.5** | **0.007775** | 0.258083 |  | 0.666475 | 0.489428 |
|  | **1.0** | **0.001851** | 0.077005 | 0.666475 |  | 0.796174 |
|  | **2.0** | **0.001234** | 0.077005 | 0.489428 | 0.796174 |  |

**S3 Table 6. Results Statistical Analysis W_max_** Lubrication 10%. Resulting p-values from statistical analysis (Mann-Whitney U-test, 5% significance level).

|  | v [mm/s] | | | | | |
| --- | --- | --- | --- | --- | --- | --- |
| v [mm/s] |  | **0.1** | **0.25** | **0.5** | **1.0** | **2.0** |
|  | **0.1** |  | 0.666475 | 1.000000 | 0.863307 | 1.000000 |
|  | **0.25** | 0.666475 |  | 0.730440 | 0.863307 | 0.386508 |
|  | **0.5** | 1.000000 | 0.730440 |  | 0.863307 | 0.931427 |
|  | **1.0** | 0.863307 | 0.863307 | 0.863307 |  | 0.730440 |
|  | **2.0** | 1.000000 | 0.386508 | 0.931427 | 0.730440 |  |

**Snap**

**S3 Table 7. Results Statistical Analysis Snap** Lubrication 90%, EID 25-28 mm. Resulting p-values from statistical analysis (Mann-Whitney U-test, 5% significance level).

|  | v [mm/s] | | | | | |
| --- | --- | --- | --- | --- | --- | --- |
| v [mm/s] |  | **0.1** | **0.25** | **0.5** | **1.0** | **2.0** |
|  | **0.1** |  | 0.050309 | **0.001234** | **0.000041** | **0.000041** |
|  | **0.25** | 0.050309 |  | 0.093912 | **0.000041** | **0.000041** |
|  | **0.5** | **0.001234** | 0.093912 |  | **0.002756** | **0.000082** |
|  | **1.0** | **0.000041** | **0.000041** | **0.002756** |  | **0.031469** |
|  | **2.0** | **0.000041** | **0.000041** | **0.000082** | **0.031469** |  |

**S3 Table 8. Results Statistical Analysis Snap** Lubrication 50%, EID 25-28 mm. Resulting p-values from statistical analysis (Mann-Whitney U-test, 5% significance level).

|  | v [mm/s] | | | | | |
| --- | --- | --- | --- | --- | --- | --- |
| v [mm/s] |  | **0.1** | **0.25** | **0.5** | **1.0** | **2.0** |
|  | **0.1** |  | **0.024434** | **0.000082** | **0.000041** | **0.000041** |
|  | **0.25** | **0.024434** |  | **0.024434** | **0.000494** | **0.000041** |
|  | **0.5** | **0.000082** | **0.024434** |  | 0.113492 | **0.007775** |
|  | **1.0** | **0.000041** | **0.000494** | 0.113492 |  | 0.161497 |
|  | **2.0** | **0.000041** | **0.000041** | **0.007775** | 0.161497 |  |

**S3 Table 9. Results Statistical Analysis Snap** Lubrication 10%, EID 25-28 mm. Resulting p-values from statistical analysis (Mann-Whitney U-test, 5% significance level).

|  | v [mm/s] | | | | | |
| --- | --- | --- | --- | --- | --- | --- |
| v [mm/s] |  | **0.1** | **0.25** | **0.5** | **1.0** | **2.0** |
|  | **0.1** |  | **0.000082** | **0.000041** | **0.000041** | **0.000041** |
|  | **0.25** | **0.000082** |  | **0.007775** | **0.000041** | **0.000041** |
|  | **0.5** | **0.000041** | **0.007775** |  | **0.001234** | **0.000041** |
|  | **1.0** | **0.000041** | **0.000041** | **0.001234** |  | **0.039984** |
|  | **2.0** | **0.000041** | **0.000041** | **0.000041** | **0.039984** |  |
